# Proteins comparison through probabilistic optimal structure local alignment

**DOI:** 10.3389/fgene.2014.00302

**Published:** 2014-09-02

**Authors:** Giovanni Micale, Alfredo Pulvirenti, Rosalba Giugno, Alfredo Ferro

**Affiliations:** ^1^Department of Computer Science, University of PisaPisa, Italy; ^2^Department of Clinical and Molecular Biomedicine, University of CataniaCatania, Italy

**Keywords:** structure comparison, protein comparison, local alignment, protein families, motifs identification, binding sites identification

## Abstract

Multiple local structure comparison helps to identify common structural motifs or conserved binding sites in 3D structures in distantly related proteins. Since there is no best way to compare structures and evaluate the alignment, a wide variety of techniques and different similarity scoring schemes have been proposed. Existing algorithms usually compute the best superposition of two structures or attempt to solve it as an optimization problem in a simpler setting (e.g., considering contact maps or distance matrices). Here, we present PROPOSAL (PROteins comparison through Probabilistic Optimal Structure local ALignment), a stochastic algorithm based on iterative sampling for multiple local alignment of protein structures. Our method can efficiently find conserved motifs across a set of protein structures. Only the distances between all pairs of residues in the structures are computed. To show the accuracy and the effectiveness of PROPOSAL we tested it on a few families of protein structures. We also compared PROPOSAL with two state-of-the-art tools for pairwise local alignment on a dataset of manually annotated motifs. PROPOSAL is available as a Java 2D standalone application or a command line program at http://ferrolab.dmi.unict.it/proposal/proposal.html.

## 1. Introduction

Protein function is commonly deduced by sequence analysis. On the other hand, most protein interactions, such as catalytic activity or gene regulation (transcription, maturation, etc.), depend on sub-regions of their 3D structures, called structural or binding motifs. Havranek and Baker (2009) show that the identification of protein-DNA interactions can help discover placements for the protein backbone. This contributes to identify the desired position and interaction of the side-chain atoms, which are responsible for protein function.

Since the structure of many proteins is still unknown and proteins with similar structural motifs often exhibit similar biological properties even when they are distantly related, 3D structure comparison can help characterize the role of many proteins. As stated in Eidhammer et al. (2000), there is no best way to make the comparison or to evaluate the alignments. Since no notion of common ancestor exists, there is a huge variety of plausible relatedness models. Forthermore, from an algorithmic standpoint, 3D structure comparison is an NP-hard problem (Goldman et al., 1999). Structural comparison is usually performed by local alignments since these are more sensitive than the global ones. Indeed, proteins with dissimilar folds may share common binding sites or interfaces. Some of them start from a specified motif (called template) in a query protein structure and search for similarities in a reference set of 3D structures.

MolLoc (Angaran et al., 2009) is a web server for comparing known binding sites, cavities or user-defined sets of residues of two or more molecular surfaces. The algorithm builds a structural alignment maximizing the extension of surface superposition. MultiBind (Shatsky et al., 2006; Peleg et al., 2008) recognizes common spatial chemical binding patterns in a set of proteins by solving a 3D k-partite matching problem through efficient geometric hashing techniques. MAPPIS (Peleg et al., 2007, 2008) relies on a similar algorithm and performs multiple alignment of protein-protein interfaces, predicting hot spot residues that contribute to the conserved patterns of the interactions. LabelHash (Moll et al., 2010), in a preprocessing phase, builds reference hash sets to guarantee instant lookup of partial motif matches. Then, these latter are expanded using a variant of the match augmentation algorithm (Chen et al., 2007). In general, the matching task can be performed with a few algorithmic techniques, such as linear programming (Lancia et al., 2001; Wohlers et al., 2009), dynamic programming (Orengo and Taylor, 1996; Jung and Lee, 2000; Ye and Godzik, 2003), depth-first searching (Stark and Russell, 2003; Ausiello et al., 2005; Chen et al., 2007), graph theory (Jambon et al., 2003; Spriggs et al., 2003; Hofbauer et al., 2004; Huan et al., 2006; Weskamp et al., 2007; Najmanovich et al., 2008; Konc and Janezic, 2010), geometric hashing (Bachar et al., 1993; Wallace et al., 1997; Shatsky et al., 2006; Moll et al., 2010), Markov chains and Monte Carlo methods (Holm and Sander, 1993; Kawabata, 2003) and combinatorial optimization (Shindyalov and Bourne, 1998; Bertolazzi et al., 2010).

Other approaches align two protein structures with no information about the location of potentially conserved binding sites. Among these we have ProBiS (Konc and Janezic, 2010, 2012) which solves the problem by making use of a maximum clique algorithm; SMAP (Xie and Bourne, 2008; Xie et al., 2009), a software package which includes a method to characterize protein structures using geometric potential, and a sequence order independent profile-profile alignment tool (SOIPPA); DaliLite (Holm and Park, 2000; Holm et al., 2008) which computes optimal and suboptimal structural alignments, by optimizing a scoring function given by the weighted sum of similarities of intramolecular distances.

To establish alignment quality several similarity scoring schemes exist. Among these the most used are the Root Mean Square Deviation (RMSD) of the optimal rigid-body superposition (Kabsch, 1976), the distance map similarity (Holm and Sander, 1993) and the Contact Map Overlap (CMO) (Lancia et al., 2001; Di Lena et al., 2010).

In this paper, we present PROPOSAL (PROteins comparison through Probabilistic Optimal Structure local ALignment), a stochastic algorithm for local alignment of 3D protein structures. PROPOSAL relies on Markov Chain Monte Carlo in connection to a Gibbs Sampling strategy which has been applied to solve the multiple local sequence alignment problem (Lawrence et al., 1993) as well as the multiple protein-protein interaction network alignment (Micale et al., 2014).

We tested PROPOSAL on the J. Skolnick benchmark (Lancia et al., 2001) and a set of known manually curated motifs, taken from the Catalytic Site Atlas (CSA) (Furnham et al., 2013). Results clearly show that the algorithm is accurate and identifies many highly conserved substructures and known functional binding sites across many proteins. Given its non-deterministic nature, it is very fast even on a large number of structures. We also compared PROPOSAL with two state-of-the-art systems, ProBiS (Konc and Janezic, 2010, 2012) and SMAP (Xie and Bourne, 2008; Xie et al., 2009) in solving a pairwise local alignment problem. The results clearly show that PROPOSAL can align proteins with different degrees of sequence similarity in reasonable time, with the highest precision.

A Java 2D standalone application with the integration of JMol for 3D visualization of alignments is freely available for download at the following URL http://ferrolab.dmi.unict.it/proposal/proposal.html, along with a command line version of PROPOSAL and a complete user documentation.

## 2. Materials and methods

Let *P* = {*P*_1_, *P*_2_,…, *P_N_*} be a set of *N* 3D protein structures and let *w* be a positive integer, with *w* ≥ 3. The goal of local protein structure alignment is to find *N* substructures of *w* residues, one for each protein, such that structure similarity is locally maximized. We call *w* the size of the local alignment.

PROPOSAL is able to find approximate solutions to the problem through a greedy and stochastic technique, by using a Markov Chain Monte Carlo (MCMC) in connection to Gibbs sampling (Geman and Geman, 1984).

PROPOSAL is an iterative method. In each iteration it tries to find an optimal local alignment of size *w*, starting from a predefined triplet of amino acids (e.g., AAC), called fingerprint. Since the fingerprint changes at every iteration and there are 20 amino acids, the maximum number of iterations performed by PROPOSAL has been set to 20^3^ = 8000.

A single iteration consists of three phases. In the first one, called *bootstrap phase*, Gibbs sampling is used to find a local alignment of *N* substructures (one for each protein), composed by 3 residues each. These substructures, called *seeds* of the alignment, represent small potential conserved motifs shared by the *N* 3D protein structures.

The quality of the seeds alignment is quantified according to a proper scoring scheme based on the average Root Mean Square Deviation (RMSD) between the aligned substructures, considering all possible pairs of proteins. The best alignments will have the lowest average RMSD.

Let *C* = {*C*_1_, *C*_2_,…, *C_k_*} and *D* = {*D*_1_, *D*_2_,…, *D_k_*} be two sets of residues. The RMSD between *C* and *D* is given by the root mean-square deviation of the Cα atomic coordinates of residues, after performing an optimal rigid body superposition. The RMSD is defined as follows:

(1) $$\begin{array}{l} RMSD(C,D)\\ \;=\sqrt{\frac{1}{w}\sum_{i\;=\;1}^{k}\left({\left(C_{ix}-D_{ix}\right)}^{2}+{\left(C_{iy}-D_{iy}\right)}^{2}+{\left(C_{iz}-D_{iz}\right)}^{2}\right)} \end{array}$$

where *C_ix_*, *C_iy_*, *C_iz_* and *D_ix_*, *D_iy_*, *D_iz_* are the 3D coordinates of residues *C_i_* and *D_i_*, respectively, after the superposition.

We computed RMSDs using QCP (Liu et al., 2010), a recently proposed algorithm that finds the optimal alignment by using a Newton-Raphson quaternion-based method.

Each seeds alignment having average RMSD ≤1 Å is extended by adding one residue at the time, until we reach an alignment of *N* motifs, each having *w* residues. The *extension phase* is performed stochastically through Gibbs sampling.

**Figure 1:**
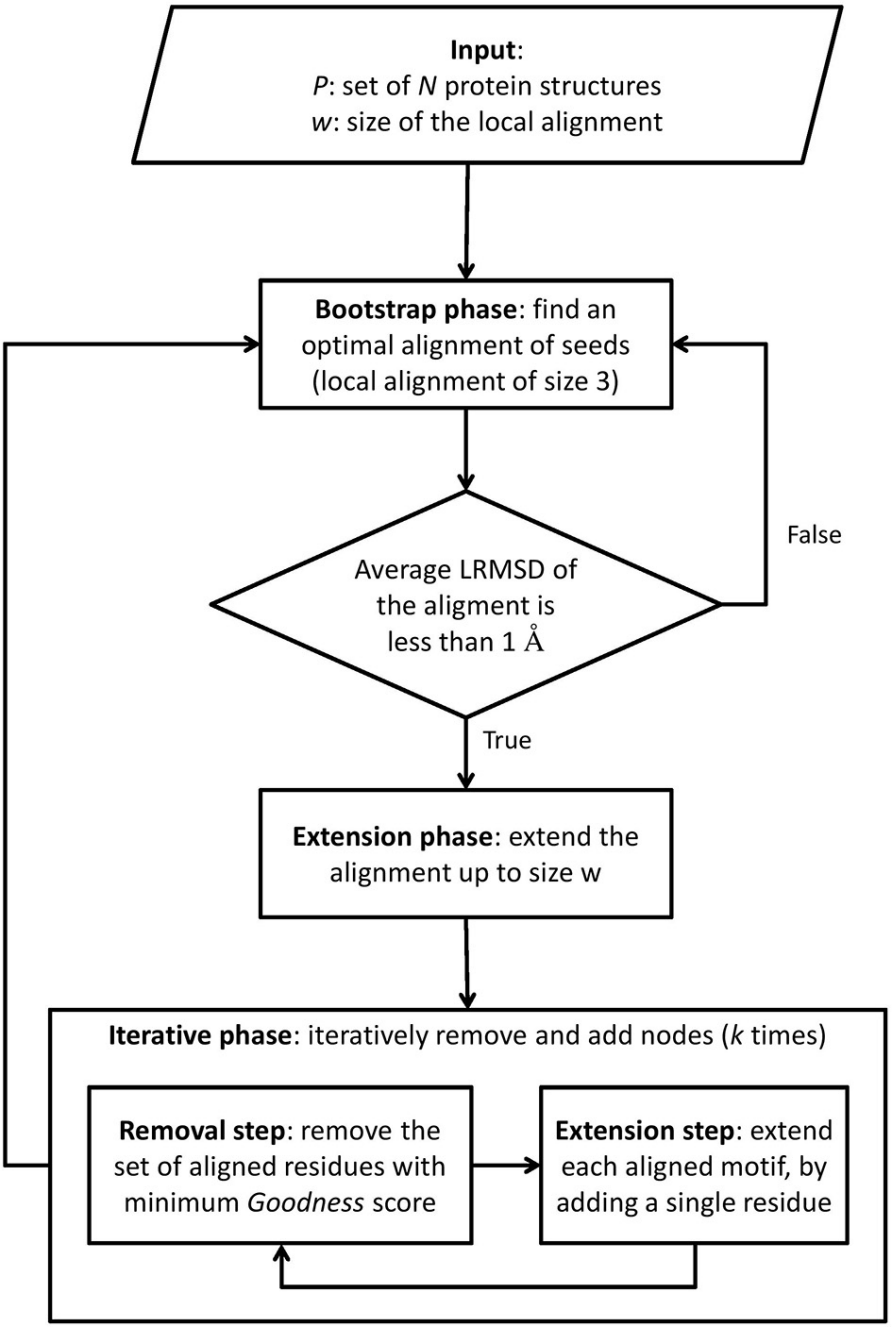
**Outline of PROPOSAL**.

Finally, in the third phase, the alignment is refined, by iteratively removing and adding single nodes to each aligned motif. This *refinement phase* produces the final local alignment (see Figure 1). The set of local alignments is then filtered by removing highly overlapping alignments.

### 2.1. Bootstrap phase

The goal of the bootstrap phase is to find an optimal alignment of small substructures of 3 nodes, called seeds. A seed is represented by a triple of residues *A* = (*A*_1_, *A*_2_, *A*_3_).

The set of possible candidates for the initial alignment consists of all seeds satisfying the following conditions:

- All residues within the seed are at distance less than 10 Å;
- The residue symbols in the triple must match the fingerprint of the corresponding iteration of PROPOSAL.

Feasible candidates are seeds satisfying both (a) and (b). If one or more proteins contain no feasible candidates, the search stops and a new iteration of PROPOSAL begins.

Once a set of suitable candidates is generated, PROPOSAL tries to construct an optimal initial alignment through Gibbs Sampling on top of a Monte Carlo Markov Chain (MCMC). In the MCMC each state represents an alignment of *N* seeds, one from each protein structure.

Starting from a random initial state (i.e., a random initial alignment), the sampling method iteratively performs a transition from a state of the chain to another, by replacing a randomly chosen seed of the current alignment with a feasible candidate of the same protein, according to a properly defined transition probability distribution. When Gibbs sampling stops, the last current alignment is returned. If the sampling procedure is iterated a sufficient number of times, it converges to a local optimum solution.

A critical task is to establish when Gibbs sampling can be stopped. The procedure ends when the alignment of seeds does not change. Let $P={\left(\frac{N-1}{N}\right)}^{i}$ be the probability that a protein structure is never selected in *i* consecutive iterations of Gibbs sampling. The number of iterations of Gibbs sampling is determined by the following parameter *k*:

(2) $$k=\max\left\{k^{\prime }:{\left(\frac{N-1}{N}\right)}^{k^{\prime }}>\alpha \right\}$$

where α is a user-defined probability threshold. If the alignment does not change for *k* consecutive iterations, the Gibbs sampling is stopped. The lower is α, the more precise and slower will be the sampling procedure. Therefore, α represents a trade-off between accuracy and speed of PROPOSAL.

The transition probability is defined on top of a similarity score, based on the distances between the residues of the seeds. Let *Dist(R*_1_, *R*_2_) be the euclidean distance between the two residues *R*_1_ and *R*_2_ of a 3D structure. Given two seeds *A* = (*A*_1_, *A*_2_, *A*_3_) and *B* = (*B*_1_, *B*_2_, *B*_3_), we define the pairwise distance between *A* and *B* as:

(3) $$PairDist(A,B)=\prod_{i=1,j=1,i<j}^{3}\left|Dist\left(A_{i},A_{j}\right)-Dist\left(B_{i},B_{j}\right)\right|$$

Now, let *S* = {*S*_1_, *S*_2_, …, *S_N_*} be the alignment of seeds at the *i*-th iteration of Gibbs sampling and suppose we have to replace *S_j_* by a feasible candidate *X* of the same protein. The similarity score of *X* is defined as the inverse of the product of all pair distances between *X* and the seeds of the current alignment (except *S_j_*):

(4) $$Sim(X)=\frac{1}{\prod_{i=1,i\neq j}^{N}PairDist(X,S_{i})}$$

The transition probability is then computed by normalizing such similarity scores in [0,1].

### 2.2. Extension of the alignment

In the extension phase, the alignment of residues is extended up to size *w* by iteratively adding *N* residues to the current alignment, one from each protein.

Suppose that we start from a substructure alignment of size *w*′<*w*. The goal is to find an optimal alignment of *N* residues *R*_1_, *R*_2_,…, *R_N_*, one for each protein, and add such residues to the substructure alignment. *R_i_* must be at distance at most equal to 10 Å from residues in the corresponding current aligned substructure. At the end of this process, the alignment size will be *w*′+1.

Each extension step is performed through a Gibbs sampling strategy similar to the one used during the bootstrap phase. In the extension phase the similarity score takes into account:

- The symbol of a candidate residue;
- The distances between the candidate residue and the aligned residues of the same structure.

Let *SA* = {*SA*_1_, *SA*_2_, …, *SA_N_*} be the current alignment of size *w*′, where each *SA_i_* = {*R*_*i*,1_, *R*_*i*,2_,…, *R*_*i,w*′_} is a set of residues, and let *A^m^* = {*A^m^*_1_, *A^m^*_2_,…, *A^m^_N_*} be the alignment of candidate residues at the generic *m*-th iteration of Gibbs sampling.

Suppose we replace *A^m^_j_* with a candidate residue *X*. First, we define a similarity score, *SimSymb(X)* which evaluates the similarity between the symbol of *X* and the symbols of residues in *A^m^* (except *A^m^_j_*):

(5) $$SimSymb(X)=\prod_{k=1,i\neq j}^{N}\;\text{SIMMATRIX}\left(X,A_{k}^{m}\right)$$

where SimMatrix(*X,A^m^_k_*) is a BLOSUM similarity score between *X* and *A^m^_k_*.

Then, we define another similarity function, *SimDist(X)*:

(6) $$SimDist(X)=\frac{1}{\prod_{k=1,k\neq j}^{N}PairDist\left(X,A_{k}^{m}\right)}$$

where *PairDist(X)* is defined as follow:

(7) $$PairDist\left(X,A_{k}^{m}\right)=\prod_{h=1}^{w^{\prime }}\left|Dist\left(X,R_{j,h}\right)-Dist\left(A_{k}^{m},R_{k,h}\right)\right|$$

Finally, the similarity score of *X*, *Sim(X)*, is the product of *SimSymb(X)* and *SimDist(X)*. Again, the transition probability of *X* is the normalization of *Sim(X)* in [0,1].

### 2.3. Refinement phase

The goal of the refinement phase is to increase the quality of the discovered alignment. An alignment of residues is iteratively removed from the current alignment of substructures and replaced with a new one. The number of iterations is bounded by a user-defined parameter called *IterRefine*. According to our experimental results (Section 3.2), a good accuracy can be achieved with relatively small values of such parameter (e.g., 10).

The replaced alignment is chosen according to a *Badness* function defined below.

Let *SA* = {*SA*_1_, *SA*_2_, …, *SA_N_*} be the final alignment of size *w*, where each *SA_i_* = {*R*_*i*,1_, *R*_*i*,2_,…, *R_i,w_*} is a set of residues. We can view the alignment *SA* as a matrix *R[N,w]*, where each column represents an alignment of residues and *R[i,j]* is the *j*-th aligned residue of the *i*-th substructure. Our final goal is to compute a *Badness* score for each column of *SA* and remove the column that maximizes the *Badness* score function from *SA*.

First, given two aligned residues *R[i,k]* and *R[j,k]*, we define the function *PairDistAligned* as follows:

(8) $$\begin{array}{l} PairDistAligned(R[i,k],R[j,k])=\prod_{h=1h\neq k}^{w}|Dist(R[i,k],R[i,h])\\ \;-Dist(R[j,k],R[j,h])| \end{array}$$

The *Badness* of a generic column *k* is:

(9) $$Badness(k)=\sum_{i,j=1,i<j}^{N}PairDistAligned(R[i,k],R[j,k])$$

Once the column with the highest *Badness* score is removed, a new single extension step is performed through the Gibbs sampling procedure described in Section 2.2.

### 2.4. Filtering overlapping alignments

The alignments produced by PROPOSAL are sorted according to the average RMSD across all possible pairs of structures. This sorted list is finally post-processed to filter highly overlapping alignments. Let *SA^i^* = {*SA^i^*_1_, *SA^i^*_2_,…, *SA^i^_N_*} be the local alignment of rank *i* in the sorted list. We define *Perc(SA^i^_k_)* as the percentage of residues in the substructure *SA^i^_k_* observed in the previous *i* − 1 alignments, and *Perc(SA^i^)* as the average value of *Perc(SA^i^_k_)* across all the aligned substructures. If *Perc(SA^i^)* is above a given threshold *Overlap*, the alignment is discarded.

## 3. Results

Three different case studies have been investigated. In the first one we analyzed the performance of our method and the effects of input parameters, using the 33 structures of Skolnick's dataset benchmark (Lancia et al., 2001), a set of large protein domains which has been used in several recent studies related to structural comparison of proteins (Pulim et al., 2008; Di Lena et al., 2010).

In the second case study, we compared PROPOSAL to SMAP (Xie and Bourne, 2008; Xie et al., 2009) and ProBis (Konc and Janezic, 2010, 2012), two algorithms for local pairwise structural alignment, on a dataset of known motifs derived from the literature and taken from the Catalytic Site Atlas (CSA) (Furnham et al., 2013).

In the last case study, following the work of Moll et al. (2011), we used a subset of these CSA motifs to test PROPOSAL as a local multiple aligner.

PROPOSAL has been implemented in Java 7 and all tests have been performed with an Intel Core i7-2670 2.2 Ghz CPU with 8 GB of RAM.

PROPOSAL needs a few parameters to be set:

- *w*: the size of the final alignments;
- α: the probability which determines the number of Gibbs Sampling iterations in the bootstrap and extension phases;
- *IterRefine*: the number of iterations during the refinement phase;
- *AvgOverlap*: a threshold bounding the average overlapping percentage of alignments.

The default values of parameters have been experimentally established as follows:

- α = 0.05;
- *IterRefine* = 10.

Both α and *IterRefine* parameters have been chosen to guarantee an optimal trade-off between speed and accuracy.

### 3.1. Tests on Skolnick dataset

The dataset is divided into four categories, depending on similarity degree and sequence length. Table 1 synthesizes the features of each family with respect to the number of proteins, the average sequence length and the average similarity.

**Table 1:** **Skolnick's dataset families**.

**Family**	**Proteins**	**Avg_seq_length**	**Avg_similarity (%)**
Flavodoxin-like fold CheY-related	8	124	15–30
Ferritin	6	170	7–70
Plastocyanin	8	99	35–90
TIM Barrel	11	250	30–90

To evaluate the reliability of PROPOSAL we considered different values of *w*, depending on proteins sequence similarity. We chose *w* = 10 for the CheY-related proteins' family, *w* = 12 for the Ferritin family, *w* = 15 for the Plastocyanin proteins, and *w* = 20 for the TIM Barrel family. In all experiments, we set *AvgOverlap = 50%* to reduce the final set of alignments. Table 2 gives the running time of PROPOSAL and the RMSD of the best alignments.

**Table 2:** **Running time and LRMSD of the best alignments on Skolnick's dataset**.

**Family**	**W**	**Running_time (s)**	**Best_RMSD (Å)**
Flavodoxin-like fold CheY-related	10	33.95	1.539
Ferritin	12	46.102	0.428
Plastocyanin	15	135.936	0.575
TIM Barrel	20	1542.929	0.428

The best alignments have been generated through the 2D alignment of their contact maps. A protein contact map is a 2D matrix storing the distances between all possible amino acids pairs of a 3D protein structure. It is represented as a graph where nodes are amino acids and edges connect nodes having a distance less than a fixed cut-off, usually 7–12 Å. A contact map is a signature of a protein structure with respect to its 3D coordinates (Vassura et al., 2008).

**Figure 2:**
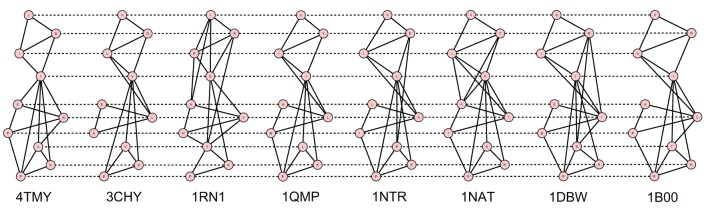
**Best alignment of the 8 Flavodoxin-like fold CheY-related contact maps with *W* = 10**.

**Figure 3:**
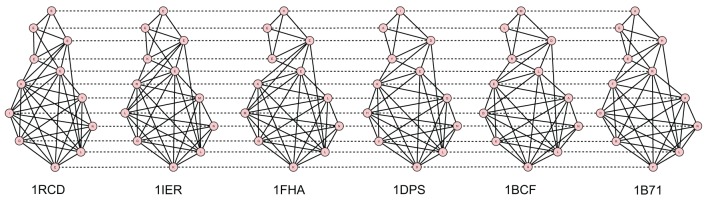
**Best alignment of the 6 Ferritin contact maps with *W* = 12**.

**Figure 4:**
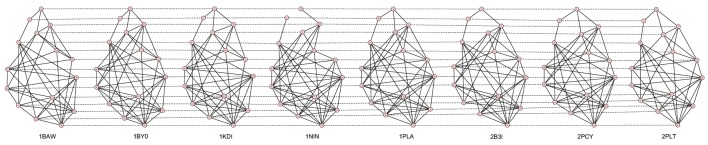
**Best alignment of the 8 Plastocyanin contact maps with *W* = 15**.

**Figure 5:**
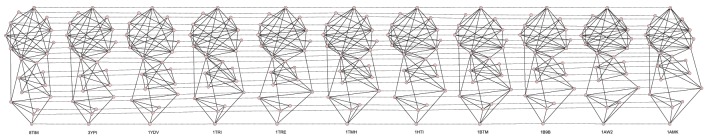
**Best alignment of the 11 TIM Barrel contact maps with *W* = 20**.

Figures 2–5 show the 10 Å cut-off contact map alignments. It can be seen that a good structural correspondence between proteins is guaranteed even when the value of *w* increases. In most cases the absence of few edges or the presence of new links between nodes are due to pairs of residues whose distance is very close to the cut-off.

**Figure 6:**
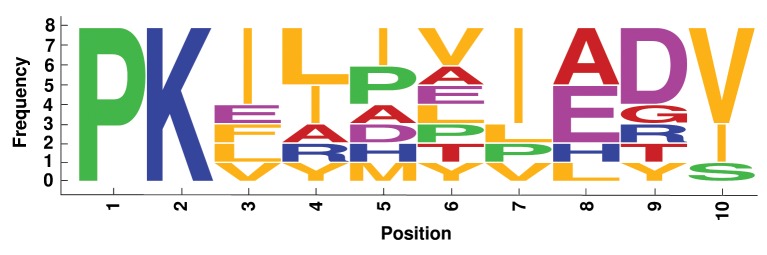
**Sequence logo of mapped residues in the best alignment of the 8 Flavodoxin-like fold CheY-related proteins**.

**Figure 7:**
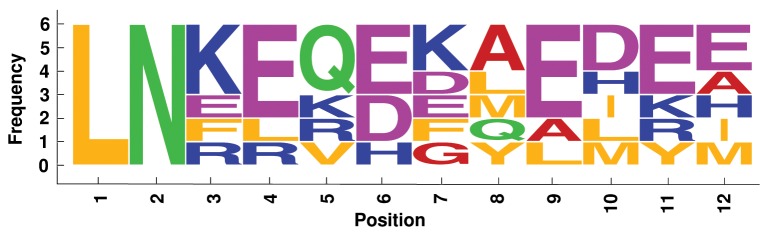
**Sequence logo of mapped residues in the best alignment of the 6 Ferritin proteins**.

**Figure 8:**
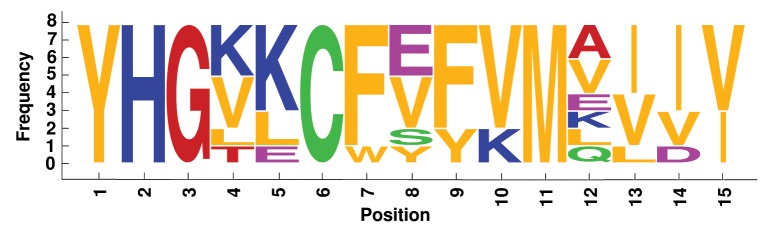
**Sequence logo of mapped residues in the best alignment of the 8 Plastocyanin proteins**.

**Figure 9:**
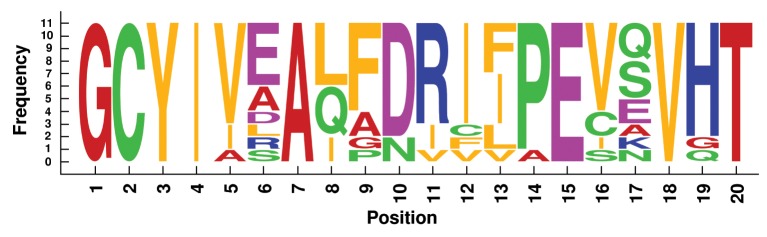
**Sequence logo of mapped residues in the best alignment of the 11 TIM Barrel proteins**.

We analyzed label similarity of the four best alignments, by building the sequence logos (Crooks et al., 2004) of mapped residues (Figures 6–9). Each position contains a graphical representation of the frequencies of residues in that position within the final mapping. Amino acids are represented with different colors, depending on their chemical properties: basic residues (K, R, H) are colored in blue, the acidic ones (D, E) in purple, the neutral ones (Q, N, P, S, C) in green, the hydrophobic ones (V, L, I, W, F, M, Y) in orange, and the remaining ones (G, T, A) in red.

Sequence logos reflect the average sequence similarity of proteins within each family: Plastocyanin and TIM Barrel proteins show the best label correspondence. The alignment of Ferritin proteins is quite interesting, since the structural similarity is high, the average LRMSD is very low (0.428 Å, Table 2), but the corresponding sequence logo shows remarkable dissimilarities between mapped residues. This is an example confirming that protein structural similarity and protein sequence similarity are not always related.

**Figure 10:**
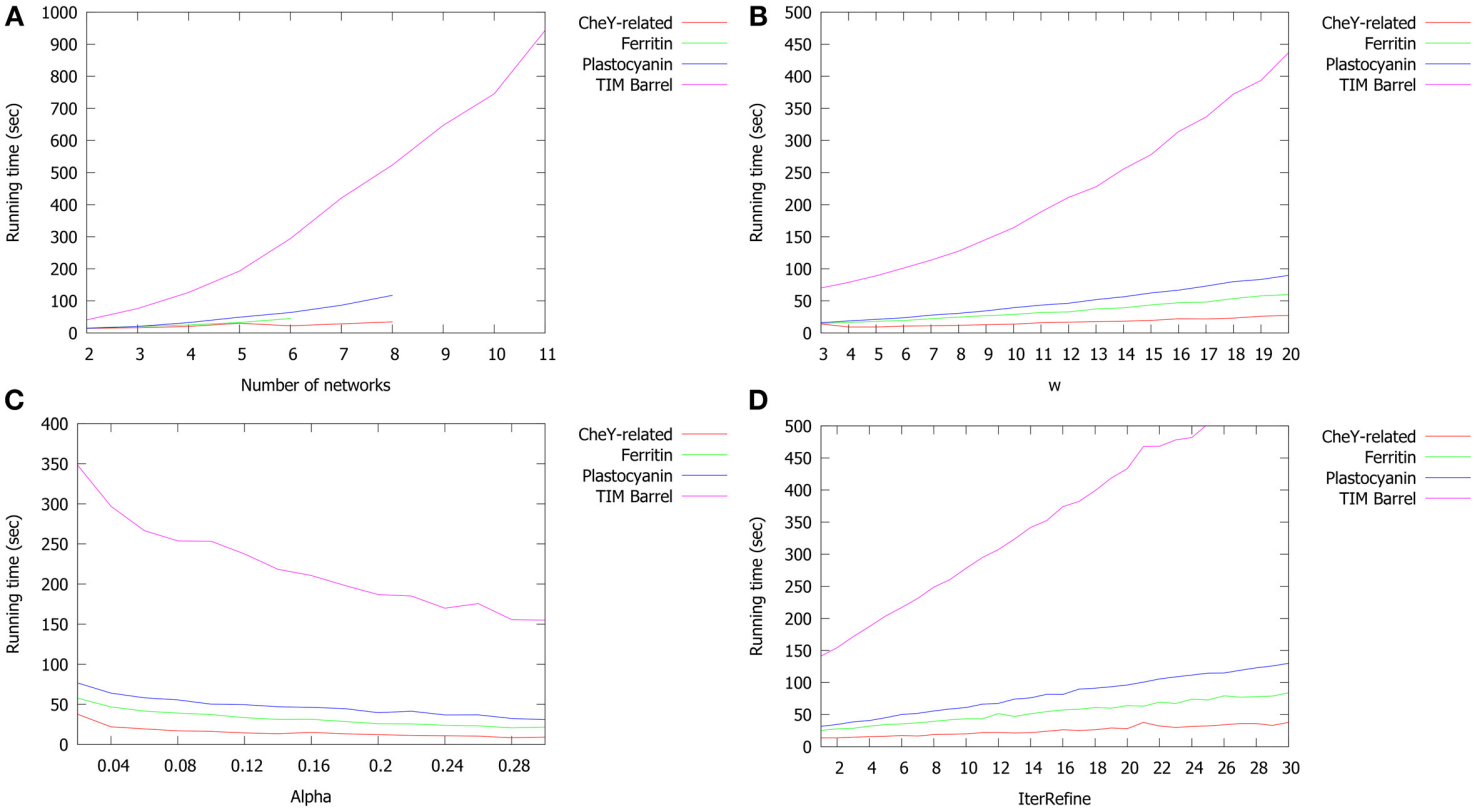
**Running time of PROPOSAL as a function of (A) number of proteins (N); (B) w; (C) α; (D) iterRefine**. Default values: *N* = 6, *w* = 15, α = 0.05, *IterRefine = 10*.

Next, we investigated the effects of varying PROPOSAL parameters. The default values are *N* = 6, *w* = 15, α = 0.05, and *IterRefine = 10*. First, we analyse how parameters influence the running time (Figure 10) by varying one parameter and leaving the rest unchanged. Figure 10A depicts the running time varying the number *N* of structures. Figure 10B deals with the effect of varying *w* from 1 to 20. Figure 10C reports the PROPOSAL behavior with α ranging from 0.01 to 0.30. Finally, in Figure 10D different values of *IterRefine* (from 1 to 30) are considered. As expected, when *N* and *w* grow and *alpha* decreases, the running time goes up. Such a trend is even more evident in the TIM Barrel family which has the highest average protein sequence length and similarity.

**Figure 11:**
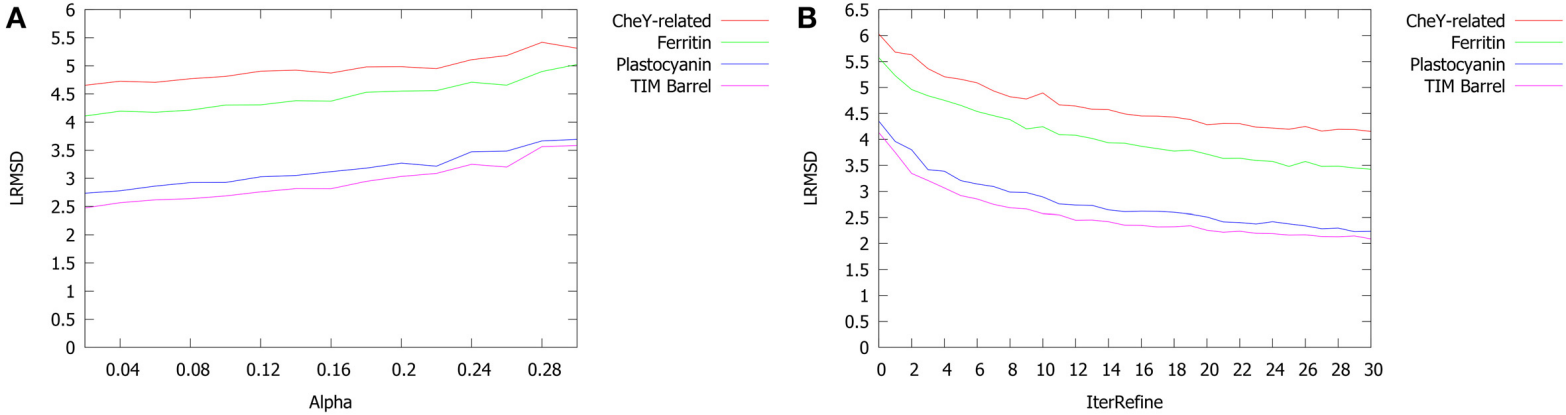
**Average LRMSD of the alignments returned by PROPOSAL on varying (A) α and (B) IterRefine**. Default values: *N* = 6, *w* = 15, α = 0.05, *IterRefine = 10*.

Figure 11 shows the influence of α and *IterRefine* on the global accuracy of PROPOSAL. We measured the average RMSD over all the computed alignments. In Figure 11A *alpha* varies from 0.01 to 0.30 and *IterRefine* is set to 10, while in Figure 11B *iterRefine* varies from 1 to 30 and α is set to 0.05. Default values (*w* = 15 and *N* = 6) were assigned. As expected, the best performance of our method are obtained with low values of α and high values of *IterRefine*. However, if we also consider the influence of such parameters on running time (in particular the *IterRefine* parameter), the best trade-off between speed and accuracy can be achieved with 0.01 ≤ α ≤ 0.1 and *IterRefine* = 10.

### 3.2. Tests on pairwise alignments

As far as we are concerned, PROPOSAL is the first algorithm proposed for multiple local alignments of protein structures. On the other hand a few existing tools can solve the pairwise local structure alignment problem (Holm and Park, 2000; Xie and Bourne, 2008; Konc and Janezic, 2010). According to the experiment results reported in Konc and Janezic (2010) and Moll et al. (2011), ProBiS and SMAP seem to be the best existing pairwise local structure alignment methods.

In order to compare PROPOSAL with ProBiS and SMAP, we run all the algorithms on a properly defined dataset of pairwise alignments.

First of all, we collected a set of 346 non-redundant literature derived small query motifs (having 4-6 residues), taken from CSA (Catalytic Site Atlas) (Furnham et al., 2013). CSA is a database of hand-annotated entries, containing enzyme active sites (i.e., a set of residues thought to be directly involved in the reaction catalyzed by an enzyme). The complete list of these motifs and the corresponding PDB structures is available in the supplementary material Table S1.

Then, we used LabelHash, which is the state-of-the-art tool for substructure matching, to search for a match between each query motif and the rest of the dataset. Finally we selected all matches with *RMSD* ≤ 1.5 Å. This resulted in a final reference dataset of 6380 pairwise alignments (the dataset is available in the supplementary material Table S2).

The dataset has many highly dissimilar pairs of proteins. In order to analyse the sequence similarity between the 6380 couples of proteins with the lowest RMSD alignments, we run BLAST and considered the percentage of residues with positive matches in the shortest sequence. We call *PPos* the latter measure. Among the 6380 couples, 3835 (≃ 60%) have *PPos* < 5% and 6173 (≃ 97%) have *PPos* < 15%.

For each couple, we run PROPOSAL with no overlapping filter (*AvgOverlap* = 100%) and *w* equals to the number of residues of the query motif. We ran SMAP and ProBiS with default parameter values.

**Figure 12:**
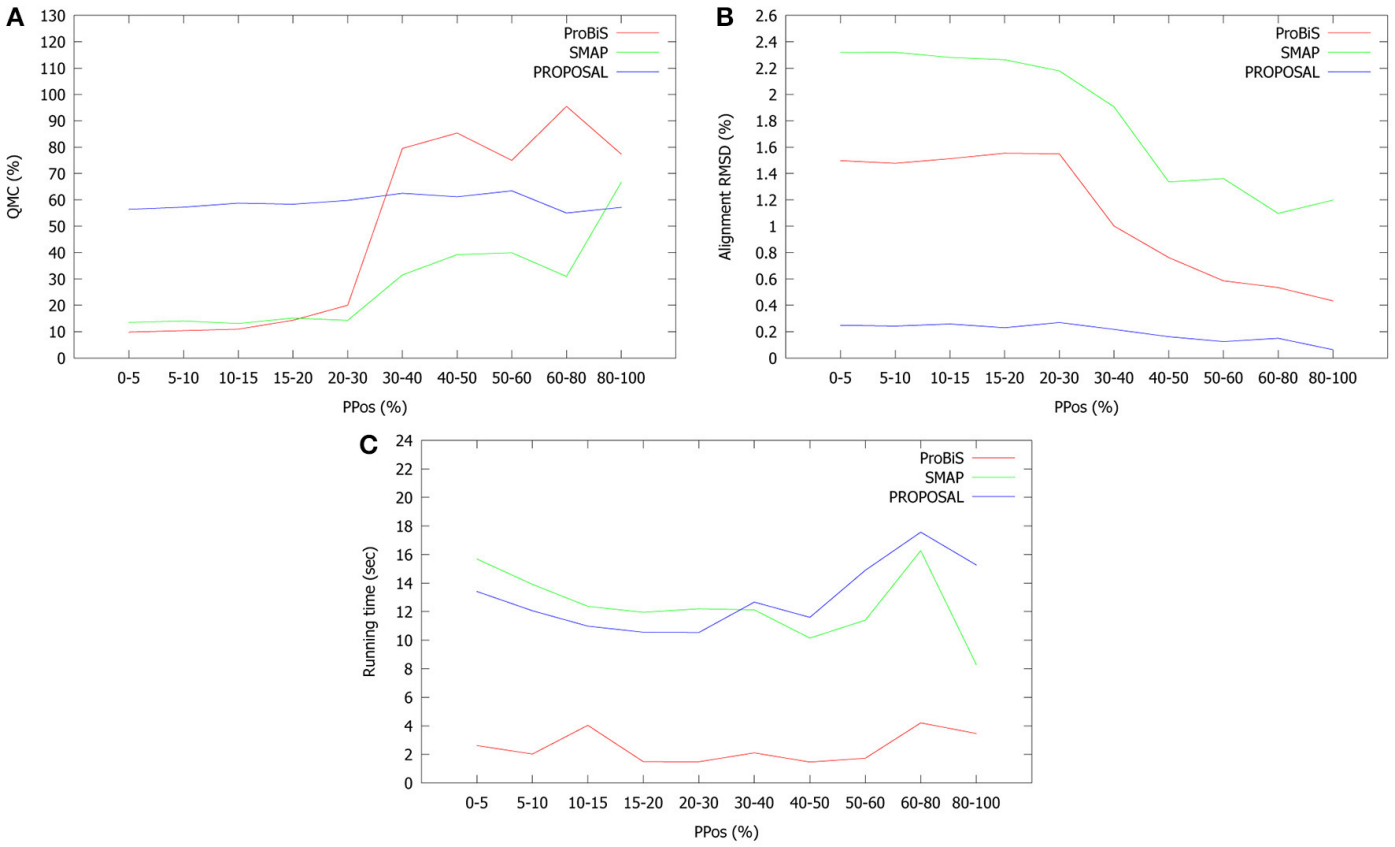
**Average (A) highest QMC, (B) corresponding alignment RMSD and (C) running time of PROPOSAL, ProBiS, and SMAP for different ranges of *PPos* similarity values**.

We analyzed the performance of the three methods on the 6380 pairwise alignments, by taking into account three parameters:

- Query motif coverage (QMC): the highest percentage of residues of the query motif which are present in an alignment returned by each algorithm;
- RMSD of the alignment with highest QMC;
- Running time;

We analyzed the average values of these parameters by considering different ranges of *PPos* similarities. All results are plotted in Figure 12.

PROPOSAL exhibits the highest QMC for highly dissimilar proteins, while for medium and high *PPos* similarities ProBiS is the best method (Figure 12A). However, in all the tested instances PROPOSAL yields the lowest average RMSD with respect to both ProBiS and SMAP. Furthermore, the difference between RMSDs tends to increase as long as *PPos* decreases (Figure 12B). We also notice that the average QMC and RMSD of PROPOSAL alignments are approximately constant for all values of *PPos*, while ProBiS and SMAP seem to be quite sensitive to protein similarity.

Finally, ProBiS is by far the fastest algorithm for all possible ranges of *PPos* similarity values (Figure 12C), while PROPOSAL and SMAP have similar running times (except for 80% ≤ *PPos* ≤ 100%, where SMAP is faster). It is worth noting that our method has been designed for solving the multiple alignment problem, while ProBiS and SMAP have been efficiently implemented for comparing pairs of protein structures. Moreover, PROPOSAL and SMAP have been implemented in Java, while ProBiS has been written in C++. Interestingly, our method is faster when *PPos* ranges from 10 to 30%. However, when proteins are very dissimilar, the convergence of Gibbs sampling in the bootstrap phase may be slower. On the other hand, when proteins are very similar PROPOSAL performs more extension and refinement phases, producing more feasible alignments. A similar trend holds for ProBiS, where the best performance is obtained when *PPos* ranges from 15 to 60%.

### 3.3. Tests on multiple alignments

In the last case study, we run PROPOSAL on a different set of 172 motifs taken from CSA to test the capability of our method to detect known conserved binding sites in the multiple case (see supplementary material Table S3).

The dataset has been built by selecting literature derived motifs of proteins belonging to fully qualified EC classes with at most 25 elements. This resulted in a final set of 172 motifs, spanning 162 distinct EC classes.

EC class (Webb, 1993) is a code having the format “EC” followed by four numbers separated by periods. It denotes the type of reaction catalyzed by an enzyme. An EC class is fully qualified if all four numbers are specified (e.g., 1.1.1.149 is fully qualified, while 1.1.1 or 1.1 are not).

For each EC family, we run PROPOSAL on the set of protein structures belonging to that family. We fixed *w* equals to the number of residues in the corresponding motif and *AvgOverlap* = 100% (i.e., no overlapping filter). The remaining parameters were set up to the default values.

We filtered out all alignments with average RMSD above 1 Å, taking for each query motif the local alignment with maximum QMC. In case of ties on QMC, the alignment with minimum average RMSD was chosen. PROPOSAL successfully completed all the alignments in about 29 h, with an average QMC of 50.08% and average running time of 10 min. In Table 3 we report motifs with highest QMC and the RMSD of the corresponding alignment (see supplementary material Table S3 for the complete list of results). Results clearly show the ability to identify known motifs from scratch. Out of 172 motifs, 24 have QMC ≥ 75% and 126 have specificity ≥ 50%.

**Table 3:** **CSA motifs with QMC ≥ 75%**.

**Protein**	**EC_class**	**Motif**	**QMC (%)**	**Avg_RMSD**
1YBV	1.1.1.252	[138, 182, 164, 178]	100	0.06814209
1QRR	3.13.1.1	[183, 186, 145, 182]	75	0.032653827
1MRQ	1.1.1.149	[50, 117, 84, 55]	75	0.063025678
1GQ8	3.1.1.11	[136, 157, 113, 135]	75	0.075239285
2JXR	3.4.23.25	[215, 32, 218, 33]	75	0.088753575
1RK2	2.7.1.15	[252, 253, 255, 254]	75	0.092735469
2PGD	1.1.1.44	[187, 190, 130, 183]	75	0.119390475
1VAS	3.1.25.1	[22, 26, 23, 2]	75	0.126902935
1CZF	3.2.1.15	[180, 201, 202, 223]	75	0.15027138
1PJB	1.4.1.1	[269, 117, 95, 74]	75	0.178174017
1RPX	5.1.3.1	[185, 43, 41, 74]	75	0.222043962
1L1L	1.17.4.2	[119, 408, 419, 410]	75	0.226235418
1DB3	4.2.1.47	[134, 160, 132, 156]	75	0.252066199
1IM5	3.5.1.19	[129, 10, 133, 94]	75	0.294630848
1ODT	3.1.1.41	[181, 269, 182, 298]	75	0.40228864
1PVD	4.1.1.1	[28, 477, 114, 115]	75	0.454688806
1U5U	4.2.1.92	[137, 67, 66, 193]	75	0.613085033
1E94	3.4.25.2	[45, 33, 124, 1]	75	0.617527201
1Z9H	5.3.99.3	[110, 113, 112, 107]	75	0.677534589
1B66	4.2.3.12	[88, 42, 133, 89]	75	0.7033398
2NAC	1.2.1.2	[284, 146, 313, 332]	75	0.78435381
1QTN	3.4.22.61	[258, 360, 350, 317]	75	0.798793943
1P4R	2.1.2.3	[431, 267, 592, 266]	75	0.936798151
1BWZ	5.1.1.7	[217, 73, 208, 159]	75	0.941959894

*Each motif is represented as a list of residue ids of the corresponding reference protein*.

In Torrance et al. (2005); Moll et al. (2010), authors observed that the EC-class coverage of a motif has not been considered for the design of CSA. Consequently, some motifs may be not conserved across all proteins in an EC class. This may be the origin of failures of PROPOSAL on the alignment tasks with QMC <50%). In some cases CSA motifs could contain one or more residues with few global matches. Moreover, two motifs could match mutually exclusive sets of proteins within the corresponding EC class. These cases may cause a drastic increase of average RMSD for that specific motif. Examples of such CSA motifs are reported in Moll et al. (2010). In order to overcome these problems, methods like Geometric Sieving (Chen et al., 2007) can be applied to refine a given motif and increase sensitivity while keeping high specificity values.

## 4. Discussion

PROPOSAL is a stochastic algorithm for local alignment of 3D protein structures relying on Markov Chain Monte Carlo in connection to a Gibbs Sampling strategy. PROPOSAL is a parameter-based algorithm. In our experimental analysis on the Skolnick's dataset (see Section 3.1) we showed that the most critical ones are α and *IterRefine*, because these influence both speed and accuracy. The best trade-off is achieved with α ranging from 0.01 to 0.1 and *IterRefine* set to 10. Therefore, default values for the algorithm are set to α = 0.05 and *IterRefine* = 10. The running time of PROPOSAL on Skolnick's dataset resulted sublinear (with respect to the number of proteins, *w*, α and *IterRefine*) for family of proteins with low and medium similarity (CheY-related, Ferritin and Plastocyanin) and linear for highly similar and long proteins (TIM Barrel).

Since PROPOSAL is the first multiple structure local alignment method, we compared it with two pairwise local alignment algorithms (ProBiS and SMAP) on a dataset of couples of query motifs and target proteins (see Section 3.2). The accuracy of PROPOSAL is defined by the highest percentage of residues of the query motif which are present in an alignment returned by each algorithm (query motif coverage), together with the quality of the alignment (RMSD score).

PROPOSAL strongly outperforms the other methods on the quality of the alignments, independently of proteins' similarity. Concerning the coverage, it is constant on proteins' similarity, whereas SMAP and ProBiS have low coverage for dissimilar proteins. However, ProBiS is 5 times faster than PROPOSAL and SMAP.

Finally, we run PROPOSAL as a multiple aligner on a subset of the above query motifs (see Section 3.3). Once again, PROPOSAL yields high quality alignments with coverage scores comparable to those obtained in the pairwise local case. Experiments also show that PROPOSAL is a valuable alternative algorithm to both identify new motifs and refine existing ones.

### Conflict of interest statement

The authors declare that the research was conducted in the absence of any commercial or financial relationships that could be construed as a potential conflict of interest.
